# Supplementary material for: Foggia Prostate Cancer Risk Calculator 2.0: A Novel Risk Calculator including MRI and Bladder Outlet Obstruction Parameters to Reduce Unnecessary Biopsies
Source: Int J Mol Sci. 2023 Jan 26;24(3):2449. doi: 10.3390/ijms24032449 (PMC9917125; doi:10.3390/ijms24032449)
Supplement: Supplementary file 1 [file ijms-24-02449-s001.zip › ijms-2158431-supplementary.pdf]

Supplementary Figure S1. Any GG PCa and GG≥2 PCa detection rates according to PIRADS score.

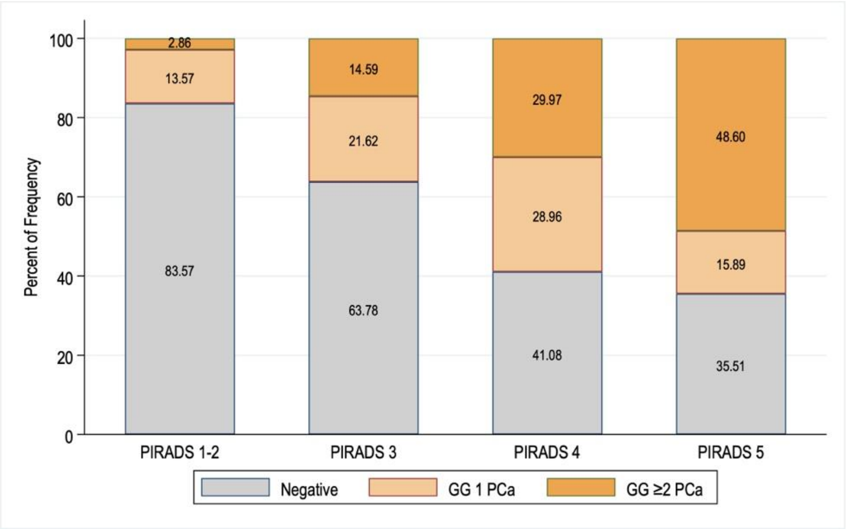

Supplementary table S1. Coefficients of the Logit function for the calculation of individual PCa and csPCa risk.

	Multivariable model predicting Any GG PCa				Multivariable model predicting GG≥2 PCa			
	**Coef.**	**95% CI**		**P>|z|**	**Coef.**	**95% CI**		**P>|z|**
**Age**	0.054	0.029	0.078	**<0.001**	0.064	0.035	0.093	**<0.001**
**Biopsy History**								
Biopsy naive	Ref.				Ref.			
Previous Negative	-0.922	-1.305	-0.539	**<0.001**	-0.910	-1.395	-0.425	**<0.001**
**DRE**								
Negative	Ref.							
Suspicious	0.303	-0.057	0.662	0.099	0.704	0.290	1.117	**0.001**
**PIRADS**								
1-2	Ref.							
3	1.137	0.566	1.709	**<0.001**	1.692	0.576	2.808	**0.003**
4	1.814	1.280	2.347	**<0.001**	2.334	1.275	3.393	**<0.001**
5	1.612	0.955	2.269	**<0.001**	2.681	1.560	3.802	**<0.001**
**PSA**	0.046	0.014	0.079	**0.005**	0.086	0.048	0.125	**<0.001**
**Prostate volume**	-0.023	-0.031	-0.016	**<0.001**	-0.024	-0.034	-0.014	**<0.001**
**PVR**								
0-50	Ref.							
>50	-0.559	-0.942	-0.176	**0.004**	-1.310	-1.865	-0.756	**<0.001**
**Costant**	-3.681	-5.305	-2.056	**<0.001**	-6.629	-8.809	-4.449	**<0.001**
